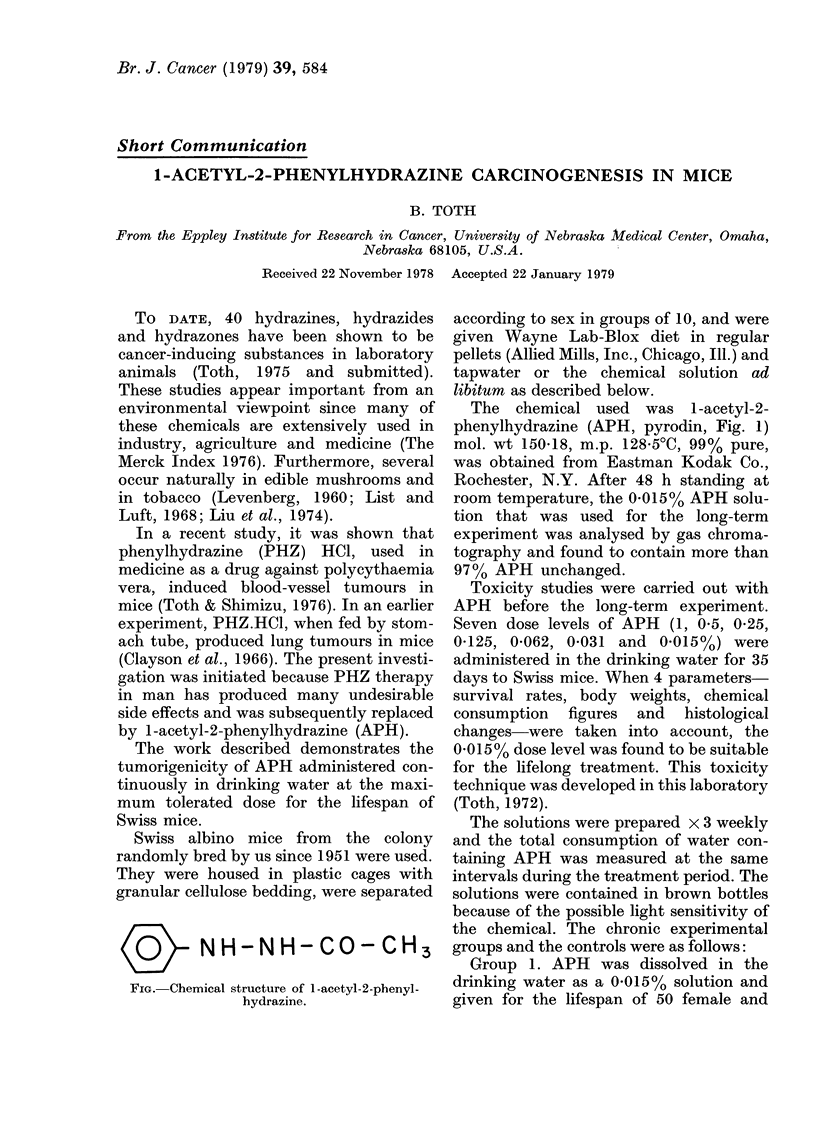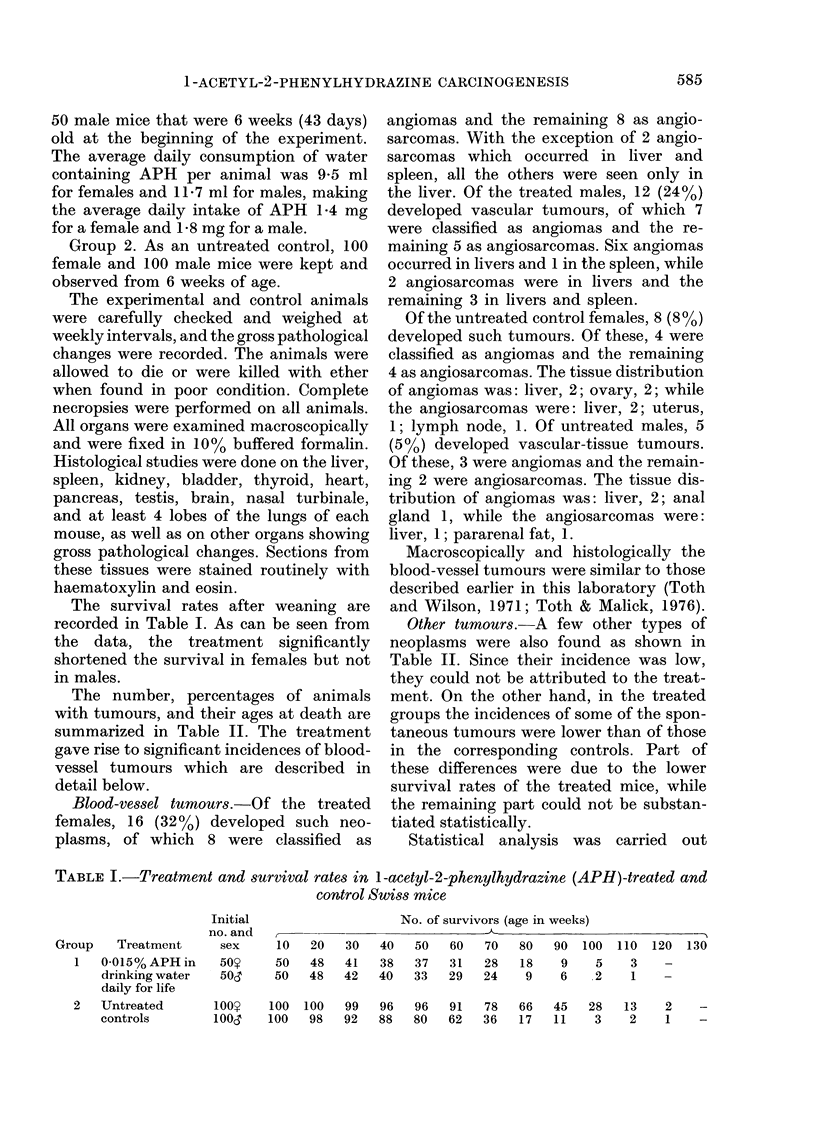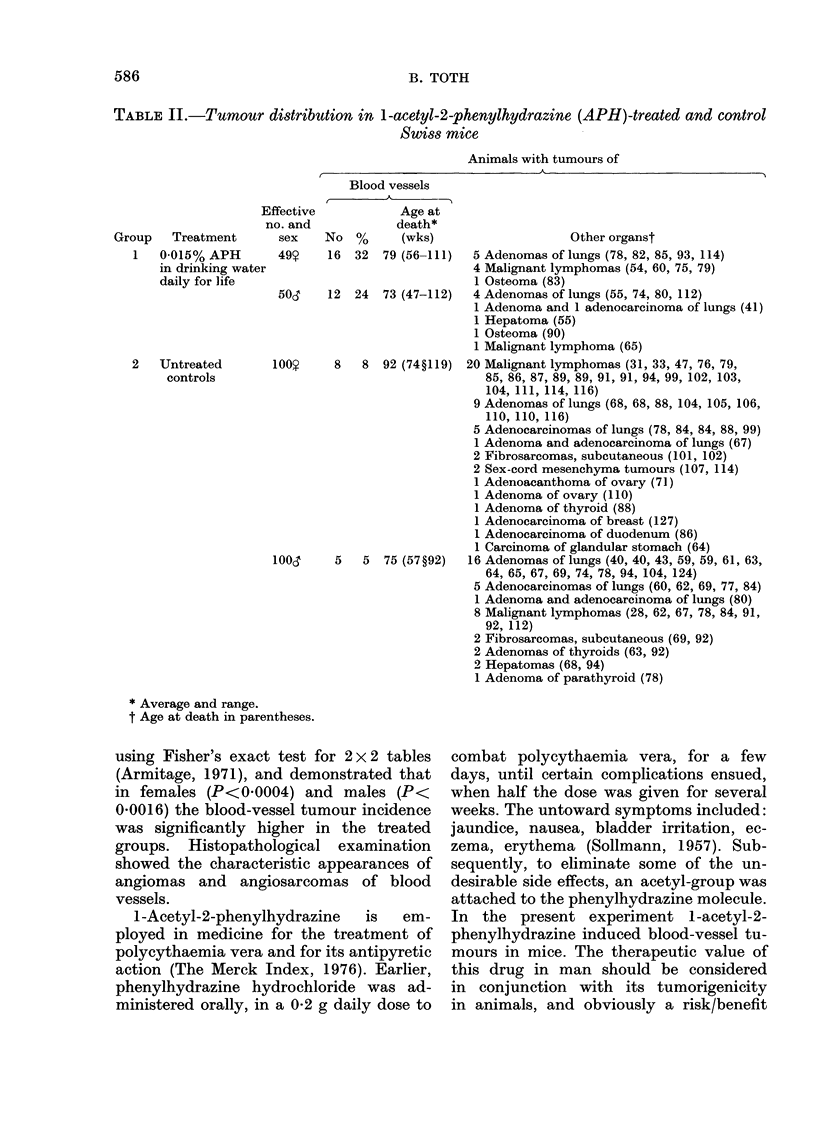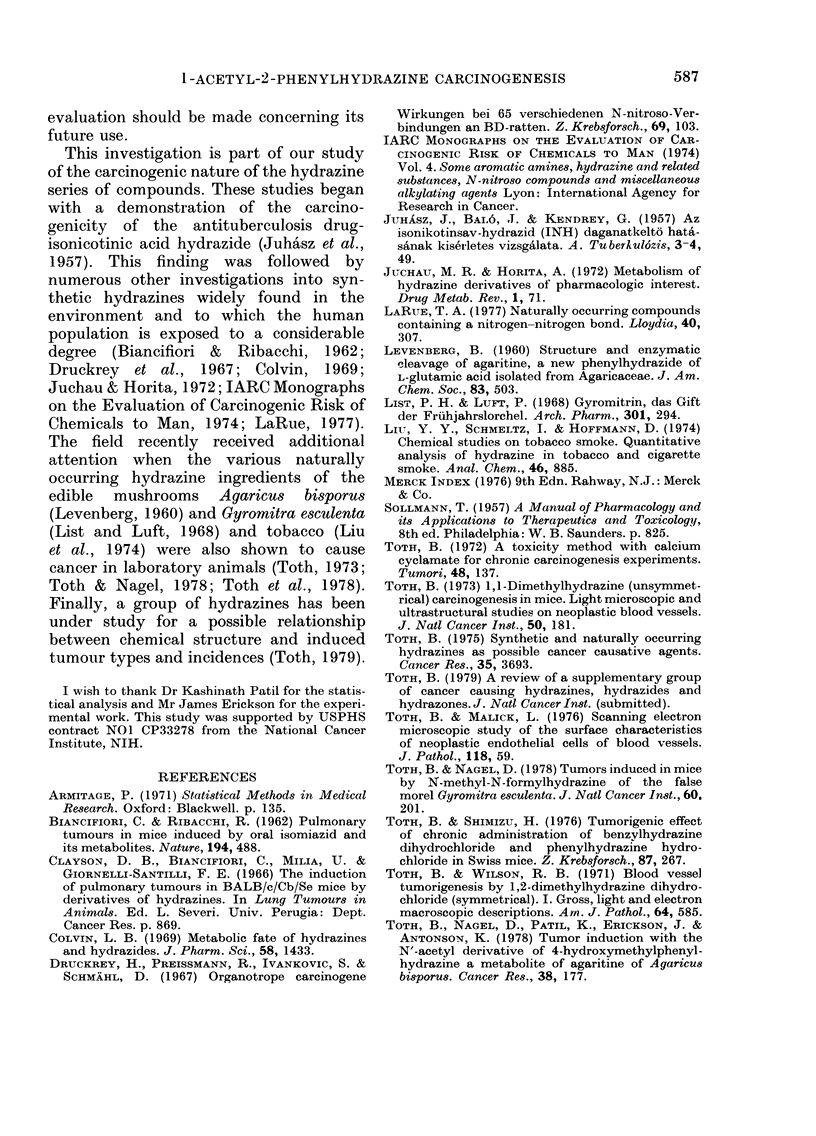# 1-Acetyl-2-phenylhydrazine carcinogenesis in mice.

**DOI:** 10.1038/bjc.1979.105

**Published:** 1979-05

**Authors:** B. Toth


*Br. J. Cancer* (1979) **39**, 584

***Short Communication***

### 1-ACETYL-2-PHENYLHYDRAZINE CARCINOGENESIS IN MICE

B. TOTH

*From the Eppley Institute for Research in Cancer, University of Nebraska Medical Center, Omaha, Nebraska* 68105, *U.S.A.*

Received 22 November 1978 Accepted 22 January 1979

TO DATE, 40 hydrazines, hydrazides and hydrazones have been shown to be cancer-inducing substances in laboratory animals (Toth, 1975 and submitted). These studies appear important from an environmental viewpoint since many of these chemicals are extensively used in industry, agriculture and medicine (The Merck Index 1976). Furthermore, several occur naturally in edible mushrooms and in tobacco (Levenberg, 1960; List and Luft, 1968; Liu *et al.*, 1974).

In a recent study, it was shown that phenylhydrazine (PHZ) HCl, used in medicine as a drug against polycythaemia vera, induced blood-vessel tumours in mice (Toth & Shimizu, 1976). In an earlier experiment, PHZ.HCl, when fed by stomach tube, produced lung tumours in mice (Clayson *et al.*, 1966). The present investigation was initiated because PHZ therapy in man has produced many undesirable side effects and was subsequently replaced by 1-acetyl-2-phenylhydrazine (APH).

The work described demonstrates the tumorigenicity of APH administered continuously in drinking water at the maximum tolerated dose for the lifespan of Swiss mice.

Swiss albino mice from the colony randomly bred by us since 1951 were used. They were housed in plastic cages with granular cellulose bedding, were separated according to sex in groups of 10, and were given Wayne Lab-Blox diet in regular pellets (Allied Mills, Inc., Chicago, Ill.) and tapwater or the chemical solution *ad libitum* as described below.

FIG.—Chemical structure of 1-acetyl-2-phenylhydrazine.

The chemical used was 1-acetyl-2-phenylhydrazine (APH, pyrodin, Fig. 1) mol. wt 150·18, m.p. 128·5°C, 99% pure, was obtained from Eastman Kodak Co., Rochester, N.Y. After 48 h standing at room temperature, the 0·015% APH solution that was used for the long-term experiment was analysed by gas chromatography and found to contain more than 97% APH unchanged.

Toxicity studies were carried out with APH before the long-term experiment. Seven dose levels of APH (1, 0·5, 0·25, 0·125, 0·062, 0·031 and 0·015%) were administered in the drinking water for 35 days to Swiss mice. When 4 parameters—survival rates, body weights, chemical consumption figures and histological changes—were taken into account, the 0·015% dose level was found to be suitable for the lifelong treatment. This toxicity technique was developed in this laboratory (Toth, 1972).

The solutions were prepared ×3 weekly and the total consumption of water containing APH was measured at the same intervals during the treatment period. The solutions were contained in brown bottles because of the possible light sensitivity of the chemical. The chronic experimental groups and the controls were as follows:

Group 1. APH was dissolved in the drinking water as a 0·015% solution and given for the lifespan of 50 female and

50 male mice that were 6 weeks (43 days) old at the beginning of the experiment. The average daily consumption of water containing APH per animal was 9·5 ml for females and 11·7 ml for males, making the average daily intake of APH 1·4 mg for a female and 1·8 mg for a male.

Group 2. As an untreated control, 100 female and 100 male mice were kept and observed from 6 weeks of age.

The experimental and control animals were carefully checked and weighed at weekly intervals, and the gross pathological changes were recorded. The animals were allowed to die or were killed with ether when found in poor condition. Complete necropsies were performed on all animals. All organs were examined macroscopically and were fixed in 10% buffered formalin. Histological studies were done on the liver, spleen, kidney, bladder, thyroid, heart, pancreas, testis, brain, nasal turbinale, and at least 4 lobes of the lungs of each mouse, as well as on other organs showing gross pathological changes. Sections from these tissues were stained routinely with haematoxylin and eosin.

The survival rates after weaning are recorded in Table I. As can be seen from the data, the treatment significantly shortened the survival in females but not in males.

The number, percentages of animals with tumours, and their ages at death are summarized in Table II. The treatment gave rise to significant incidences of blood-vessel tumours which are described in detail below.

*Blood-vessel tumours.*—Of the treated females, 16 (32%) developed such neoplasms, of which 8 were classified as angiomas and the remaining 8 as angiosarcomas. With the exception of 2 angiosarcomas which occurred in liver and spleen, all the others were seen only in the liver. Of the treated males, 12 (24%) developed vascular tumours, of which 7 were classified as angiomas and the remaining 5 as angiosarcomas. Six angiomas occurred in livers and 1 in the spleen, while 2 angiosarcomas were in livers and the remaining 3 in livers and spleen.

Of the untreated control females, 8 (8%) developed such tumours. Of these, 4 were classified as angiomas and the remaining 4 as angiosarcomas. The tissue distribution of angiomas was: liver, 2; ovary, 2; while the angiosarcomas were: liver, 2; uterus, 1; lymph node, 1. Of untreated males, 5 (5%) developed vascular-tissue tumours. Of these, 3 were angiomas and the remaining 2 were angiosarcomas. The tissue distribution of angiomas was: liver, 2; anal gland 1, while the angiosarcomas were: liver, 1; pararenal fat, 1.

Macroscopically and histologically the blood-vessel tumours were similar to those described earlier in this laboratory (Toth and Wilson, 1971; Toth & Malick, 1976).

*Other tumours.*—A few other types of neoplasms were also found as shown in Table II. Since their incidence was low, they could not be attributed to the treatment. On the other hand, in the treated groups the incidences of some of the spontaneous tumours were lower than of those in the corresponding controls. Part of these differences were due to the lower survival rates of the treated mice, while the remaining part could not be substantiated statistically.

Statistical analysis was carried out

TABLE I.—*Treatment and survival rates in 1-acetyl-2-phenylhydrazine (APH)-treated and control Swiss mice*

| Group | Treatment | Initial no. and sex | No. of survivors (age in weeks) 10 | 20 | 30 | 40 | 50 | 60 | 70 | 80 | 90 | 100 | 110 | 120 | 130 |
|---|---|---|---|---|---|---|---|---|---|---|---|---|---|---|---|
| 1 | 0·015% APH in drinking water daily for life | 50♀ | 50 | 48 | 41 | 38 | 37 | 31 | 28 | 18 | 9 | 5 | 3 | – | |
| | | 50♂ | 50 | 48 | 42 | 40 | 33 | 29 | 24 | 9 | 6 | 2 | 1 | – | |
| 2 | Untreated controls | 100♀ | 100 | 100 | 99 | 96 | 96 | 91 | 78 | 66 | 45 | 28 | 13 | 2 | – |
| | | 100♂ | 100 | 98 | 92 | 88 | 80 | 62 | 36 | 17 | 11 | 3 | 2 | 1 | – |

TABLE II.—*Tumour distribution in 1-acetyl-2-phenylhydrazine (APH)-treated and control Swiss mice*

| | | | Animals with tumours of | | | |
|---|---|---|---|---|---|---|
| | | | Blood vessels | | | |
| Group | Treatment | Effective no. and sex | No | % | Age at death* (wks) | Other organs† |
| 1 | 0·015% APH in drinking water daily for life | 49♀ | 16 | 32 | 79 (56–111) | 5 Adenomas of lungs (78, 82, 85, 93, 114) |
| | | | | | | 4 Malignant lymphomas (54, 60, 75, 79) |
| | | | | | | 1 Osteoma (83) |
| | | 50♂ | 12 | 24 | 73 (47–112) | 4 Adenomas of lungs (55, 74, 80, 112) |
| | | | | | | 1 Adenoma and 1 adenocarcinoma of lungs (41) |
| | | | | | | 1 Hepatoma (55) |
| | | | | | | 1 Osteoma (90) |
| | | | | | | 1 Malignant lymphoma (65) |
| 2 | Untreated controls | 100♀ | 8 | 8 | 92 (74§119) | 20 Malignant lymphomas (31, 33, 47, 76, 79, 85, 86, 87, 89, 89, 91, 91, 94, 99, 102, 103, 104, 111, 114, 116) |
| | | | | | | 9 Adenomas of lungs (68, 68, 88, 104, 105, 106, 110, 110, 116) |
| | | | | | | 5 Adenocarcinomas of lungs (78, 84, 84, 88, 99) |
| | | | | | | 1 Adenoma and adenocarcinoma of lungs (67) |
| | | | | | | 2 Fibrosarcomas, subcutaneous (101, 102) |
| | | | | | | 2 Sex-cord mesenchyma tumours (107, 114) |
| | | | | | | 1 Adenoacanthoma of ovary (71) |
| | | | | | | 1 Adenoma of ovary (110) |
| | | | | | | 1 Adenoma of thyroid (88) |
| | | | | | | 1 Adenocarcinoma of breast (127) |
| | | | | | | 1 Adenocarcinoma of duodenum (86) |
| | | | | | | 1 Carcinoma of glandular stomach (64) |
| | | 100♂ | 5 | 5 | 75 (57§92) | 16 Adenomas of lungs (40, 40, 43, 59, 59, 61, 63, 64, 65, 67, 69, 74, 78, 94, 104, 124) |
| | | | | | | 5 Adenocarcinomas of lungs (60, 62, 69, 77, 84) |
| | | | | | | 1 Adenoma and adenocarcinoma of lungs (80) |
| | | | | | | 8 Malignant lymphomas (28, 62, 67, 78, 84, 91, 92, 112) |
| | | | | | | 2 Fibrosarcomas, subcutaneous (69, 92) |
| | | | | | | 2 Adenomas of thyroids (63, 92) |
| | | | | | | 2 Hepatomas (68, 94) |
| | | | | | | 1 Adenoma of parathyroid (78) |

* Average and range.

† Age at death in parentheses.

using Fisher's exact test for 2×2 tables (Armitage, 1971), and demonstrated that in females ($P<0{\cdot}0004$) and males ($P<0{\cdot}0016$) the blood-vessel tumour incidence was significantly higher in the treated groups. Histopathological examination showed the characteristic appearances of angiomas and angiosarcomas of blood vessels.

1-Acetyl-2-phenylhydrazine is employed in medicine for the treatment of polycythaemia vera and for its antipyretic action (The Merck Index, 1976). Earlier, phenylhydrazine hydrochloride was administered orally, in a 0·2 g daily dose to combat polycythaemia vera, for a few days, until certain complications ensued, when half the dose was given for several weeks. The untoward symptoms included: jaundice, nausea, bladder irritation, eczema, erythema (Sollmann, 1957). Subsequently, to eliminate some of the undesirable side effects, an acetyl-group was attached to the phenylhydrazine molecule. In the present experiment 1-acetyl-2-phenylhydrazine induced blood-vessel tumours in mice. The therapeutic value of this drug in man should be considered in conjunction with its tumorigenicity in animals, and obviously a risk/benefit evaluation should be made concerning its future use.

This investigation is part of our study of the carcinogenic nature of the hydrazine series of compounds. These studies began with a demonstration of the carcinogenicity of the antituberculosis drug-isonicotinic acid hydrazide (Juhász *et al.*, 1957). This finding was followed by numerous other investigations into synthetic hydrazines widely found in the environment and to which the human population is exposed to a considerable degree (Biancifiori & Ribacchi, 1962; Druckrey *et al.*, 1967; Colvin, 1969; Juchau & Horita, 1972; IARC Monographs on the Evaluation of Carcinogenic Risk of Chemicals to Man, 1974; LaRue, 1977). The field recently received additional attention when the various naturally occurring hydrazine ingredients of the edible mushrooms *Agaricus bisporus* (Levenberg, 1960) and *Gyromitra esculenta* (List and Luft, 1968) and tobacco (Liu *et al.*, 1974) were also shown to cause cancer in laboratory animals (Toth, 1973; Toth & Nagel, 1978; Toth *et al.*, 1978). Finally, a group of hydrazines has been under study for a possible relationship between chemical structure and induced tumour types and incidences (Toth, 1979).

I wish to thank Dr Kashinath Patil for the statistical analysis and Mr James Erickson for the experimental work. This study was supported by USPHS contract NO1 CP33278 from the National Cancer Institute, NIH.

### REFERENCES

Armitage, P. (1971) *Statistical Methods in Medical Research*. Oxford: Blackwell. p. 135.

Biancifiori, C. & Ribacchi, R. (1962) Pulmonary tumours in mice induced by oral isomiazid and its metabolites. *Nature*, **194**, 488.

Clayson, D. B., Biancifiori, C., Milia, U. & Giornelli-Santilli, F. E. (1966) The induction of pulmonary tumours in BALB/c/Cb/Se mice by derivatives of hydrazines. In *Lung Tumours in Animals*. Ed. L. Severi. Univ. Perugia: Dept. Cancer Res. p. 869.

Colvin, L. B. (1969) Metabolic fate of hydrazines and hydrazides. *J. Pharm. Sci.*, **58**, 1433.

Druckrey, H., Preissmann, R., Ivankovic, S. & Schmähl, D. (1967) Organotrope carcinogene Wirkungen bei 65 verschiedenen N-nitroso-Verbindungen an BD-ratten. *Z. Krebsforsch.*, **69**, 103.

IARC Monographs on the Evaluation of Carcinogenic Risk of Chemicals to Man (1974) Vol. 4. *Some aromatic amines, hydrazine and related substances, N-nitroso compounds and miscellaneous alkylating agents* Lyon: International Agency for Research in Cancer.

Juhász, J., Baló, J. & Kendrey, G. (1957) Az isonikotinsav-hydrazid (INH) daganatkeltö hatásának kisérletes vizsgálata. *A. Tuberkulózis*, **3–4**, 49.

Juchau, M. R. & Horita, A. (1972) Metabolism of hydrazine derivatives of pharmacologic interest. *Drug Metab. Rev.*, **1**, 71.

LaRue, T. A. (1977) Naturally occurring compounds containing a nitrogen–nitrogen bond. *Lloydia*, **40**, 307.

Levenberg, B. (1960) Structure and enzymatic cleavage of agaritine, a new phenylhydrazide of L-glutamic acid isolated from Agaricaceae. *J. Am. Chem. Soc.*, **83**, 503.

List, P. H. & Luft, P. (1968) Gyromitrin, das Gift der Frühjahrslorchel. *Arch. Pharm.*, **301**, 294.

Liu, Y. Y., Schmeltz, I. & Hoffmann, D. (1974) Chemical studies on tobacco smoke. Quantitative analysis of hydrazine in tobacco and cigarette smoke. *Anal. Chem.*, **46**, 885.

Merck Index (1976) 9th Edn. Rahway, N.J.: Merck & Co.

Sollmann, T. (1957) *A Manual of Pharmacology and its Applications to Therapeutics and Toxicology*, 8th ed. Philadelphia: W. B. Saunders. p. 825.

Toth, B. (1972) A toxicity method with calcium cyclamate for chronic carcinogenesis experiments. *Tumori*, **48**, 137.

Toth, B. (1973) 1,1-Dimethylhydrazine (unsymmetrical) carcinogenesis in mice. Light microscopic and ultrastructural studies on neoplastic blood vessels. *J. Natl Cancer Inst.*, **50**, 181.

Toth, B. (1975) Synthetic and naturally occurring hydrazines as possible cancer causative agents. *Cancer Res.*, **35**, 3693.

Toth, B. (1979) A review of a supplementary group of cancer causing hydrazines, hydrazides and hydrazones. *J. Natl Cancer Inst.* (submitted).

Toth, B. & Malick, L. (1976) Scanning electron microscopic study of the surface characteristics of neoplastic endothelial cells of blood vessels. *J. Pathol.*, **118**, 59.

Toth, B. & Nagel, D. (1978) Tumors induced in mice by N-methyl-N-formylhydrazine of the false morel *Gyromitra esculenta*. *J. Natl Cancer Inst.*, **60**, 201.

Toth, B. & Shimizu, H. (1976) Tumorigenic effect of chronic administration of benzylhydrazine dihydrochloride and phenylhydrazine hydrochloride in Swiss mice. *Z. Krebsforsch.*, **87**, 267.

Toth, B. & Wilson, R. B. (1971) Blood vessel tumorigenesis by 1,2-dimethylhydrazine dihydrochloride (symmetrical). I. Gross, light and electron macroscopic descriptions. *Am. J. Pathol.*, **64**, 585.

Toth, B., Nagel, D., Patil, K., Erickson, J. & Antonson, K. (1978) Tumor induction with the N′-acetyl derivative of 4-hydroxymethylphenylhydrazine a metabolite of agaritine of *Agaricus bisporus*. *Cancer Res.*, **38**, 177.